# The Metabolic Control of Myeloid Cells in the Tumor Microenvironment

**DOI:** 10.3390/cells10112960

**Published:** 2021-10-30

**Authors:** Eloise Ramel, Sebastian Lillo, Boutaina Daher, Marina Fioleau, Thomas Daubon, Maya Saleh

**Affiliations:** 1 ImmunoConcEpT, CNRS, University of Bordeaux, UMR 5164, F-33000 Bordeaux, France; eramel@immuconcept.org (E.R.); sebastian.lillo@u-bordeaux.fr (S.L.); mfioleau@immuconcept.org (M.F.); 2 Institut de Biochimie et Génétique Cellulaires (IBGC), CNRS, University of Bordeaux, UMR 5095, F-33000 Bordeaux, France; boutaina.daher@u-bordeaux.fr (B.D.); thomas.daubon@u-bordeaux.fr (T.D.); 3 Department of Medicine, McGill University, Montreal, QC H3G 0B1, Canada

**Keywords:** cellular metabolism, immunometabolism, myeloid cells, macrophages, tumor microenvironment, cancer, immunotherapy

## Abstract

Myeloid cells are a key determinant of tumor progression and patient outcomes in a range of cancers and are therefore being actively pursued as targets of new immunotherapies. The recent use of high-dimensional single-cell approaches, e.g., mass cytometry and single-cell RNA-sequencing (scRNA-seq) has reinforced the predominance of myeloid cells in the tumor microenvironment and uncovered their phenotypic diversity in different cancers. The cancerous metabolic environment has emerged as a critical modulator of myeloid cell functions in anti-tumor immunity versus immune suppression and immune evasion. Here, we discuss mechanisms of immune-metabolic crosstalk in tumorigenesis, with a particular focus on the tumor-associated myeloid cell’s metabolic programs. We highlight the impact of several metabolic pathways on the pro-tumoral functions of tumor-associated macrophages and myeloid-derived suppressor cells and discuss the potential myeloid cell metabolic checkpoints for cancer immunotherapy, either as monotherapies or in combination with other immunotherapies.

## 1. Introduction

Macrophages are key regulators of tissue homeostasis and acquire niche-dependent programming that results in distinct phenotypes and functions across tissues [1]. In tumors, myeloid cells, including tumor-associated macrophages (TAMs) and myeloid-derived suppressor cells (MDSCs), represent a predominant immune population with significant heterogeneity. It is well established that these innate immune cells greatly influence cancer growth and metastasis, and they are currently being intensely studied as next generation targets of cancer immunotherapies [2]. Towards this effort, much work is devoted to precisely mapping the myeloid cell heterogeneity and landscapes in tumors, using single-cell approaches, in order to identify subsets with potent immunosuppressive and tumor-promoting properties. Not only are myeloid cells highly versatile in response to different stimuli in the tumor microenvironment (TME), e.g., heterogeneous oxygen distribution, acidity, lactate, etc., their ontogeny greatly contributes to their phenotypes and functions [3]. For instance, embryonically derived tissue resident macrophages (TRMs) exhibit distinct profiles and activities in tumorigenesis than bone marrow-derived TAMs [4,5,6].

In order to target deleterious myeloid cells in the TME, an interesting and promising entry point is to modulate their cellular metabolism to re-program them into anti-tumoral immune effectors with a cytotoxic capacity. Similarly to highly proliferative cancer cells that prefer aerobic glycolysis over mitochondrial oxidative phosphorylation (OXPHOS) to support their growth and proliferation (the Warburg effect) [7], pro-inflammatory and cytotoxic TAMs rely on aerobic glycolysis. Immunosuppressive cells, on the other hand, use OXPHOS and fatty acid oxidation (FAO), as reviewed in [8], which no longer holds and thus requires a re-evaluation. The bulk of the work characterizing macrophage immunometabolism pathways has been conducted using in vitro polarization of bone marrow-derived macrophages (BMDMs) into two extreme phenotypes referred as M1 (pro-inflammatory) and M2 (anti-inflammatory) cells; however, these do not reflect the heterogeneity of TAMs and MDSCs in vivo, and their metabolic requirements in the context of nutrient deprivation and metabolite competition among different cells of the TME. In this review, we focus on recent studies exploring the immunometabolism of macrophages in vivo, particularly in the context of the cancerous TME. We describe the metabolic crosstalk between myeloid cells, cancer cells, and other cells in the TME and highlight new findings on the myeloid cell metabolism that diverge from the M1/M2 immunometabolism paradigms.

## 2. The Role of Hypoxia, Aerobic Glycolysis and Lactate in the Recruitment and Functions of TAMs and MDSCs

Hypoxia is an environmental factor with a great impact on the regulation of myeloid cell profiles within the TME. In hypoxic conditions, hypoxia-induced factors (HIF)-1*α* and HIF-2*α* accumulate and engage several signaling pathways that converge on phenotypic and functional changes in myeloid cells. In 2010, Werno C et al. examined the role of HIF-1*α* in macrophage tumor infiltration. They demonstrated that in a coculture in vitro system, a loss of HIF-1*α* in macrophages derived from cd11b^+^ splenocytes did not impact the capacity to infiltrate tumor spheroids [9]. Imtiyaz et al. generated myeloid-specific HIF-2*α* knockout mice and demonstrated that HIF-2*α* was required for TAM infiltration, potentially by upregulating the expression of CXCR4 and M-CSFR [10]. A follow-up study by Casazza A et al. reported the context-dependent role of HIF-2*α* in the infiltration of TAMs, particularly in the hypoxic areas of the tumor. Although the initial attraction of TAMs to tumors is mediated by hypoxia-induced Semaphorin 3A and Neuropilin-1 (Nrp1) signaling, these cells then become “entrapped” via a secondary suppression of Nrp1 by HIF-2*α*. In such a hypoxic environment, the macrophages acquire immunosuppressive and pro-angiogenic properties that promote tumor growth. In contrast, the forced retainment of TAMs in normoxic areas, through the genetic deletion of *Nrp1*, results in their differentiation into inflammatory anti-tumoral cytotoxic macrophages [11]. This work provided early evidence that the heterogeneity of TAMs depended on their localization with respect to an oxygen gradient.

In these early studies, both HIF-1*α* [9] and HIF-2*α* [10] were shown to promote the differentiation of macrophages into pro-inflammatory and cytotoxic cells. Indeed, HIF-1*α*-deficient macrophages exhibited reduced pro-inflammatory cytokine production and impaired tumorilytic capacity [9], and myeloid-specific *Hif2a* null mice were resistant to lipopolysaccharide-induced endotoxemia [10]. These results were consistent with the reported requirement of HIF-1*α*-induced aerobic glycolysis in the activation of M1 macrophages [12,13]. Moreover, Tannahill et al. have previously demonstrated that, as a result of a break in the tricarboxylic acid cycle (TCA) cycle in M1 macrophages [14], succinate accumulates, leading to the stabilization of HIF-1*α* and driving the expression of aerobic glycolysis and inflammatory effectors such as pro-IL-1*β* [12].

**Figure 1:**
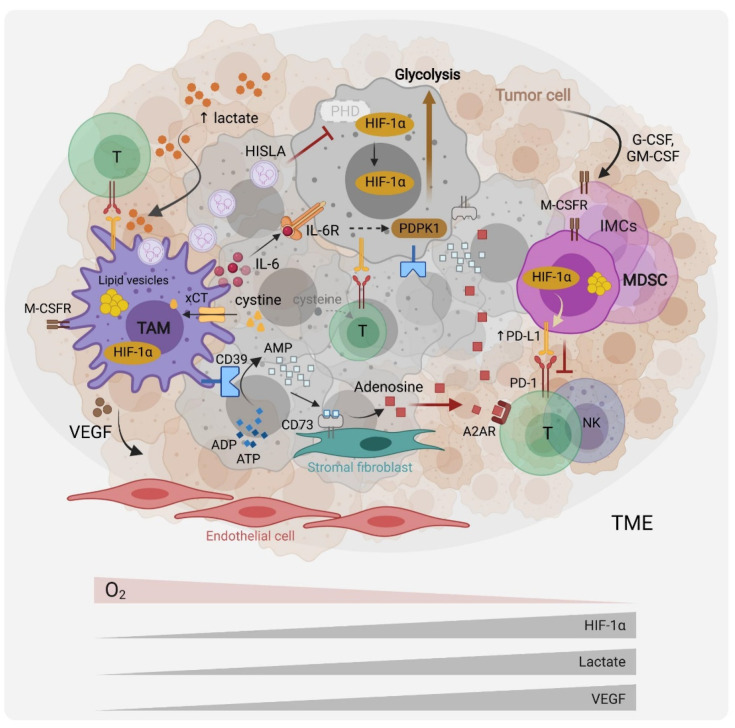
Myeloid cells metabolic interactions with other cells in the tumor microenvironment. Tumor cells produce G-CSF and GM-CSF that recruit myeloid cells, including immature myeloid cells (IMC), myeloid-derived suppressor cells (MDSCs) and tumor-associated macrophages (TAMs) to the TME. Hypoxia, which results in the stabilization of the hypoxia-induced factor (HIF)-1a, the tumor cell’s upregulation of aerobic glycolysis (the Warburg effect), and subsequent TME lactate accumulation and acidification, modulate myeloid cells towards pro-tumoral and immunosuppressive effectors. Through the expression of cytokines such as interleukin (IL)-6, immune checkpoints ligands such as Programmed Death Ligand 1 (PD-L1), and growth factors such as Vascular Endothelial Growth Factor (VEGF), myeloid cells promote tumor-cell survival, immune evasion and angiogenesis. Recently, TAMs were reported to promote the Warburg effect via IL-6 signaling as well as through vesicles containing a HIF-1a-stabilizing lncRNA (HISLA). Through the cystine transporter xCT, MDSCs and TAMs deplete the TME of cysteine, which is necessary for T cell effector functions. They further suppress T cell activity by upregulating the expression of the ectonucleotidases CD39 and CD73 at their surface, leading to adenosine production and immunosuppressive signaling via the adenosine receptor (A2AR). TAMs and MDSCs accumulate lipid droplets in cytosolic vesicles and appear to rely on lipid metabolism and fatty acid oxidation for their pro-tumoral functions.

In contrast to the M1/M2 immunometabolism paradigm, more recent studies have shown that immunosuppressive TAMs and MDSCs are highly glycolytic, and that aerobic glycolysis, HIF-1*α* activity and succinate accumulation in the TME, promote their immunosuppressive phenotypes and functions. For instance, Alexander et al. demonstrated that the myeloid-specific deletion of the circadian clock regulator *Bmal1*, led to impaired macrophage mitochondrial metabolism, the accumulation of mitochondrial reactive oxygen species (mROS) and succinate, HIF-1*α* stabilization and enhanced aerobic glycolysis. Such an aberrant HIF-1*α* activation in TAMs promoted the TAM-pro-tumoral function and tumor development [15]. Another direct evidence of the high glycolytic activity of TAMs came from work by de-Brito et al., who showed that blocking glycolysis with 2-deoxy-glucose (2-DG) in macrophages derived from human monocytes and cultured in tumor-cell-line conditioned media diminished the production of M2 markers [16]. Extracellular succinate further contributes to the migration of macrophages into the tumor site and their differentiation into tumor-promoting cells, by engaging its receptor SUCNR1 on the macrophage-cell surface, which drives the PI3K-HIF-1*α* pathway [17]. In contrast, extracellular succinate stimulates SUCNR1 on the tumor-cell surface furthering tumor proliferation and metastasis [17]. HIF-1*α* also promotes the differentiation of tumor MDSCs into more potent suppressors of T cell activity [18]. A potential mechanism was proposed by Noman et al., who identified the immune checkpoint PD-L1 as a transcriptional target of HIF-1*α* [19] (Figure 1). Collectively, HIF-1*α* potentiates aerobic glycolysis and pro-inflammatory cytokine production in an inflammatory setting. In the TME, HIF proteins drive the pro-tumoral activities of TAMs and the differentiation of MDSC into potent suppressors of anti-tumor immunity.

**Figure 2:**
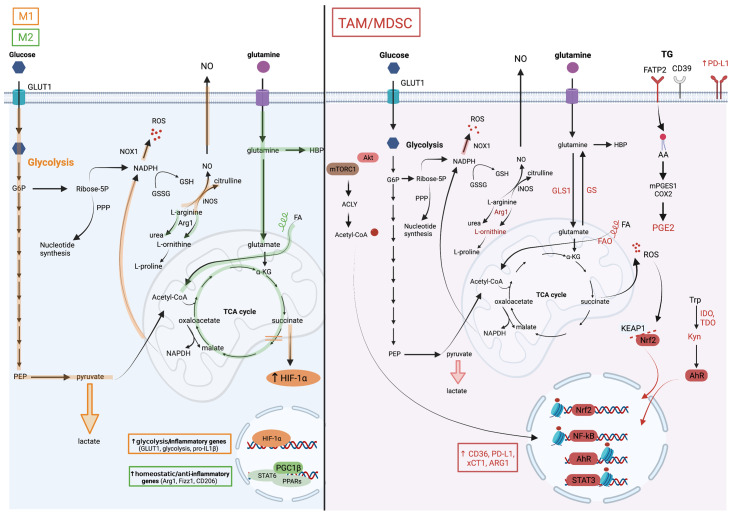
Metabolic pathways associated with TAM and MDSC pro-tumoral and myelosuppressive functions. The M1/M2 paradigm established from an in vitro macrophage culture system distinguishes two extreme macrophage metabolic phenotypes. (**On the left**), the metabolic pathways in orange are upregulated in M1 macrophages and those in green in M2 macrophages. M1 macrophages are highly glycolytic which is a consequence of HIF-1a stabilization in response to succinate accumulation due to breaks in the TCA cycle. Besides inducing the expression of glycolytic enzymes, HIF-1a also drives the production of pro-inflammatory factors such as IL-1b. In contrast, M2 macrophages favor glutamine consumption, upregulate the hexosamine biosynthetic pathway (HBP), and rely on fatty acid oxidation (FAO) for their energetic needs. STAT6 downstream of IL-4 signaling and PPARg with its co-activator PGC1b are key for their differentiation. In contrast to this simplified system, the TME complexity results in a marked myeloid cell heterogeneity with M1/M2 mixed profiles and divergent metabolic characteristics. (**On the right**), the cellular metabolic pathways upregulated in TAMs and MDSCs are illustrated. These cells are highly glycolytic but are dependent on glutamine and lipid consumption for their pro-tumoral functions. Despite their heightened aerobic glycolysis, they upregulate M2-like genes through the accumulation of acetyl coA, which is downstream of the AKT/mTOR-dependent activation of ATP Citrate Lyase (ACLY), and histone acetylation. Histone lactylation, which was reported to occur in M1 macrophages at later stages of activation and proposed as a mechanism to terminate the inflammatory response, might regulate TAM functions, but this remains to be tested. The heightened mitochondrial respiration in tumor-associated myeloid cells leads to the elevated production of reactive oxygen species (ROS). To withhold oxidative stress, they activate the transcription factor NRF2, which induces the expression of anti-oxidative genes and of the cystine transporter xCT1, among others. Myeloid cells in the TME upregulate triglycerides (TG) uptake, for instance through fatty acid transport protein (FATP)2, as reported in granulocytic 18 MDSCs, lipid accumulation in vesicles, lipolysis and FAO. Consequently, they also produce inflammatory and immunosuppressive lipid mediators such as the prostaglandin PGE2. Furthermore, they metabolize arginine and tryptophan into metabolites that favor tumor growth, including L-ornithine and L-kynurenine (Kyn). The latter is a ligand of aryl hydrocarbon receptor (AHR), which promotes myelosuppressive functions in the TME via transcriptional activity.

Besides HIF proteins, the mechanistic target of rapamycin (mTOR) exerts important functions in myeloid-cell migration, polarization and function. The mTOR exists in one of two complexes, mTOR complex (mTORC)1 or mTORC2, by associating with either Raptor or Rictor, respectively. Moreover, mTOR signaling regulates a broad set of basic cellular and metabolic processes, including translation, cell growth and proliferation [20,21], and has established roles in immunoregulation, acting in both innate and adaptive immune cells [22]. The mTOR activation in cancer cells promotes tumorigenesis through various mechanisms including the recruitment of MDSCs, by inducing the production of the myelopoiesis and mobilizing cytokine G-CSF [23]. To elucidate the specific contributions of the two mTOR complexes, mice with the myeloid-specific deletion of either *Raptor* or *Rictor* were generated. In the latter, mTORC2 was established as a regulator of M2 polarization and a negative regulator of pro-inflammatory macrophage differentiation [24]. Consistently, mice with the myeloid-specific deletion of mTORC2 were more susceptible to colitis-associated colorectal cancer (CAC) exhibiting an enhanced production of inflammatory mediators, including SPP1/osteopontin [24]. Notably, the role of mTORC1 in TAM differentiation has been controversial; Ding et al. used a subcutaneous transplantable Lewis lung carcinoma (LLC) mouse model and showed that the loss of Raptor in TAMs did not impact primary tumor growth, but enhanced lung metastasis by promoting the expansion of metastasis-associated macrophages [25]. In contrast, Collins et al. recently reported that mTORC1 controlled M2 polarization [26]. Despite impaired glycolysis, the myeloid-specific deletion of mTORC1 led to enhanced pro-inflammatory functions in vitro and in vivo, due to the enhanced histone acetylation downstream of the inhibited sirtuins [26]. This is in line with previous work by Horng and coworkers, who identified the Akt-mTORC1 pathway as a regulator of ATP-citrate lyase (ACLY) that synthesizes cytosolic acetyl CoA and triggers M2 gene induction through histone acetylation [27] (Figure 2). Consistent with a role of mTORC1 in M2 polarization, the myeloid-specific deletion of the mTORC1 inhibitor Tuberous sclerosis 2 (*Tsc2*) resulted in the constitutive activation of mTORC1, and led to the expansion of M2 cells in vivo and a sarcoidosis-like granulomatous disease [28]. In a combined approach of metabolomics, proteomics, mRNA expression analysis, and enzymatic activity measurements using *Tsc2*-deficient mice, Wilson et al. identified Phosphoglycerate Dehydrogenase (PHGDH), the first enzyme in the de novo serine/glycine biosynthesis pathway, as a central mTORC1-induced effector of anti-inflammatory macrophage differentiation [29]. Collectively, while mTOR activation was shown to promote glucose uptake and aerobic glycolysis in M1 macrophages in vitro, enhancing pro-inflammatory responses and blunting the responsiveness to IL-4 in M2 macrophages [30], it exerted pro-tumorigenic roles in TAMs, instructing an M2-like, pro-angiogenic and pro-metastatic program.

The key metabolic features of the TME include hypoxia, acidosis and lactate accumulation [31], which collectively contribute to myeloid cell infiltration and their differentiation into pro-tumoral immunosuppressive effectors. Indeed, hypoxia and lactate gradients have been shown to act as TME morphogens, eliciting the progressive differentiation of TAMs into pro-angiogenic orchestrators [32], partly through the HIF-induced production of vascular endothelial growth factor (VEGF) [33] (Figure 1). Such positional information might organize tumors into structured entities akin to the morphogenic regulation of embryonic tissue development. Mechanistically, lactate has been shown to activate mTORC1, resulting in the suppression of ATP6V0d2, a vacuolar ATPase involved in HIF-2*α* lysosomal degradation. Such a lactate-driven link between mTORC1 activation and the stabilization of HIF-2*α* favors for pro-tumoral macrophage differentiation [33]. In addition, lactate rewires the macrophage metabolism into M2 cells, independently of IL-4/IL-13 signaling [34]. Using the M1/M2 culture system and human monocyte-derived macrophages, Zhang et al. demonstrated that the recently identified lactate-derived epigenetic histone modification termed lactylation, regulated the expression of macrophage “homeostatic” genes such as arginase (ARG)1 at later stages of M1 activation, potentially to terminate the inflammatory response [35]. Whether this mechanism impacts TAM phenotypes and functions remains to be examined. Independently of lactate, acidosis can polarize macrophages into an anti-inflammatory phenotype. Bohn T et al. reported that acidosis sensing by G-protein coupled receptors (Gpr)65 and potentially Gpr132 promoted such effects via CAMP-dependent expression of the Inducible CAMP Early Repressor (ICER), also known as CAMP Responsive Element Modulator (CREM), that induced several genes associated with the M2 phenotype including *Arg1*, *Clec10a*, *Vegfa* and *Hif1a*. Consistently, the myeloid-specific deletion of *Crem1* has resulted in improved anti-tumoral immune responses and tumor rejection in a mouse model of melanoma [36].

In the cancer setting, TAMs often express an M1/M2 mixed phenotype [37], as reaffirmed recently by single-cell approaches in several cancers, including glioblastoma [38]. This has also been observed in non-cancerous settings in vivo. For example, using a retinopathy mouse model, Liu et al. showed that retinal microglia and macrophages adjacent to endothelial cells (EC) are highly glycolytic, pro-angiogenic and express both M1 and M2 markers, in response to the elevated lactate levels released by EC cells [39]. Hyper-glycolytic macrophages accumulate acetyl-coA that epigenetically drives M2 pro-angiogenic factors, which in turn promote EC sprouting and the establishment of a pathological vascular niche [39]. Two recent studies have further illustrated the metabolic crosstalk among cells in the TME favoring tumor growth. Yajuan Zhang et al. demonstrated a new pro-tumoral mechanism by which the TAMs augment the capacity of tumor cells to undergo glycolysis. By secreting IL-6, macrophages could boost the Warburg effect in cancer cells through IL-6 receptor signaling, inducing the activation of 3-phosphoinositide-dependent protein kinase 1 (PDPK1) [40]. PDPK1 phosphorylates the glycolysis enzyme phosphoglycerate kinase 1 (PGK1) steering the catabolism of 1,3-bisphosphoglycerate (1,3-BPG) into 3-phosphoglycerate (3-PG) to fuel glycolysis [40]. A second mechanism by which TAMs upregulate tumor cell glycolysis is through the secretion of extracellular vesicles that carry a HIF-1*α*-stabilizing long non-coding RNA (lncRNA) termed HISLA. Upon uptake by the tumor cell, this lncRNA disrupts the HIF-1*α* interaction with prolyl hydroxylase (PHD)2, resulting in the stabilization of HIF-1*α*. In a feed-forward loop, production of the HISLA is stimulated by tumor-cell-derived lactate [41] (Figure 1).

## 3. Amino Acids, Metabolism Controls, Myeloid Cell Phenotypes and Functions in the TME

### 3.1. Glutamine

Glutamine is a key metabolite required for several cellular processes, such as nucleotide synthesis, amino acid production and glycosylation. Glutamine is provided from exogenous intake or produced by glutamine synthetase (GS, also known as Glutamate-Ammonia Ligase [GLUL]) from glutamate and ammonia (Figure 2). Several studies have reinforced the dependency of pro-tumoral myeloid cells on glutamine metabolism.

On one hand, the genetic or pharmacological inhibition of GS in macrophages has been shown to rewire their metabolism towards a pro-inflammatory phenotype impacting the activity of macrophages in cancer metastasis in vivo. For instance, using the M1/M2 culture system, Palmieri et al. demonstrated that the inhibition of the GS-modulated macrophage metabolism and function towards an M1 profile notably led to succinate accumulation, HIF-1*α* stabilization, and to a decreased ability of IL-10-treated macrophages to suppress T cell activity, and to promote the endothelial capillary network formation [42]. These findings were reinforced by observations that the myeloid-specific deletion of *Glul (Gs)* in mice thwarted LLC lung metastasis, albeit without affecting the subcutaneous primary tumor growth [42]. More recently, and in line with Palmieri’s findings, Menga et al. identified glufosinate as a specific inhibitor of human GS and obtained similar results using different cancer metastasis mouse models. This showed that the glufosinate enhanced polarization of TAMs to an inflammatory phenotype promoted the tumor accumulation of cytotoxic T cells, normalized the vasculature and suppressed metastasis [43].

On the other hand, the interference with glutaminolysis by inhibiting glutaminase (GLS1) activity was similarly effective in blunting the immunosuppressive phenotype of macrophages, tumor-associated immature myeloid cells and MDSCs. Such a treatment increased the efficacy of the immune checkpoint blockade, highlighting the potential for inhibiting myeloid cell metabolic checkpoints for cancer immunotherapy. Initially, Liu et al. used in vitro M1/M2 cultures to demonstrate that glutaminolysis, leading to the generation of *α*KG and thus a high *α*KG/succinate ratio, promoted the metabolic and epigenetic upregulation of M2 genes via the αKG-dependent histone demethylase Jumonji d3 (jmjd3) [44]. In contrast, in M1 macrophages, *α*KG suppressed the NF-κB pathway by inhibiting IKK through PHD-dependent proline hydroxylation, which led to impaired proinflammatory responses [44]. Glutaminolysis is not only implicated in macrophage polarization, but also plays a role in the differentiation and function of immunosuppressive myeloid cells. Both Wu et al. [45] and Sun et al. [46] demonstrated the presence of hematopoietic progenitor cells (HPC) in cancers that give rise to immature myeloid cells (IMC) and MDSCs. Although these IMCs are highly glycolytic, it is not glycolysis but rather glutaminolysis that drives their expansion, regardless of glucose availability, via the production of αKG. A heightened glutamine consumption by cancer cells and subsequent glutamate accumulation in the TME was proposed as a tumor immune evasion mechanism, as glutamate signaling via the NMDA receptor stimulated M-CSFR expression and drove the IMC immunosuppressive functions [45]. Consistently, Sun et al. have shown that glutamine starvation activates the IRE1*α*-JNK pathway in mouse mammary tumor cells and enhances their production of G-CSF and GM-CSF. Subsequently, this recruits myeloid HPC from the bone marrow into the secondary lymphoid organs and the tumor (Figure 1), establishing an immunosuppressive environment [46]. To further ascertain the immunosuppressive effect of glutaminolysis, Wu et al. showed that the inhibition of GLS1 enhanced the therapeutic effect of anti-PD-L1 in a transplantable mammary carcinoma mouse model [45]. Moreover, Oh et al. corroborated the immunosuppressive and pro-tumoral effect of glutaminolysis. The authors used pharmacologic inhibitors of this pathway and showed that besides inducing the immunogenic cell death of tumor cells, these inhibitors decreased the recruitment of MDSCs and converted them to pro-inflammatory cells, enhancing the efficacy of the immune checkpoint blockade [47]. Surprisingly, the GLS1 inhibition reduced the expression of the Indoleamine 2,3-dioxygenase (IDO), an enzyme involved in the catabolism of tryptophan into the immunosuppressive metabolite L-kynurenine (Kyn) [47] (Figure 2). Together, these studies point towards the glutamine metabolism in macrophages as an important immunosuppressive pathway and a potential therapeutic target.

### 3.2. Tryptophan

It is well established that the tryptophan catabolic activity of IDO in TAMs impairs T cell activation [48]. Su et al. have demonstrated that the induction of the IDO expression in TAMs is triggered by the sensing of phagocytosed tumor DNA by the AIM2 inflammasome, linking antibody-dependent cell phagocytosis (ADCP) in macrophages to the suppression of CD8 T cells and to antibody-dependent cell cytotoxicity (ADCC) in natural killer (NK) cells [49]. AIM2-dependent IL-1*β* does not only upregulate IDO but also the PD-L1 expression leading to immunosuppression, as shown in a breast cancer mouse model. Consistently, the inhibition of IDO in combination with anti-PD-L1 potentiated the therapeutic efficacy of the anti-HER2 antibody, completely reversing the suppressive effects of ADCP and the enhanced anti-tumor immunity [49]. Kyn, the product of the IDO-derived tryptophan metabolism, has been identified as a ligand of the transcription factor aryl hydrocarbon receptor (AHR) [50] (Figure 2). In glioblastoma, Kyn produced by glioma cells activates AHR in TAMs leading to enhanced recruitment via CCR2 upregulation [51]. AHR promotes macrophage immunosuppressive functions through several mechanisms, including KLF4 upregulation of M2 gene expression, repression of NF-κB via SOCS2-TRAF6 signaling, and immunosuppressive adenosine production through the induction of the ectonucleotidase CD39 (Figure 2). Consistently, the myeloid-specific deletion of AHR results in the improved rates of mouse survival and decreases the tumor burden in response to the GL261 glioma orthotopic implantation [51]. Besides impairing the effector functions of macrophages and CD8 T cells in tumors, the AHR activation in Tregs was shown to promote the recruitment of myeloid cells to the TME and to support their immunosuppressive functions [52]. Thus, a combination therapy targeting IDO and AHR could boost the immune checkpoint blockade efficacy in IDO-overexpressing tumors.

### 3.3. Arginine

Arginine metabolism is an important determinant of macrophage phenotypes and functions. Arginine catabolism to nitric oxide (NO) and citrulline by inducible nitric oxide synthetase (iNOS) (Figure 2) is an essential pathway for inflammatory macrophage phagocytic, cytotoxic and anti-tumoral functions. Indeed, besides favoring a pro-inflammatory state in macrophages via NO [53], arginine metabolism by iNOS triggers anti-tumor immunity via citrulline. A recent report has demonstrated that citrullination enhances the immunogenicity of epitopes presented on Major Histocompatibility Complex (MHC) class II (MHC-II), leading to an improved anti-tumoral immunity against established tumors, associated with enhanced T helper (Th)1 responses, decreased infiltration of MDSCs, and a memory response upon tumor rechallenge [54]. On the other hand, arginine metabolism to ornithine, a precursor of polyamines, by ARG1 (Figure 2) is necessary for macrophage homeostatic functions and is usurped in TAMs to support tumor growth [55]. Early studies have shown that TAMs expressing high levels of ARG1 are key effectors of tumor immune evasion, and that the inhibition of ARG1 reduces the tumor growth, as demonstrated in the LLC transplantable mouse model [56]. More recently, high ARG1 activity has been shown to support the survival of immunosuppressive TIM4^+^ TAMs (human CRIg^+^ TAMs) through mitophagy, induced by the inhibition of mTOR in response to arginine depletion. Since these TAMs rely on mitochondrial OXPHOS for energetic demands, they are susceptible to oxidative damage-induced apoptosis when ARG1-dependent mitophagy is inhibited [57]. This mechanism might thus be exploited as a therapeutic strategy to counter immunosuppression in cancer. An alternative approach has been proposed by Badeux et al., who showed that a stable ARG1 enzyme, pegzilarginase, administered systemically, can starve tumors of exogenous arginine and improve anti-tumor immunity, presumably through M1 polarization [58]. This therapy showed enhanced efficacy in combination with anti-PD-L1 or agonistic anti-OX40 immunotherapy in tumor-bearing mice, supporting combination therapies in cancer patients [58].

### 3.4. Cystine, Glutamate and Oxidative Stress

Another amino acid involved in immunoregulation in the TME is cysteine. Early work has demonstrated that one of the mechanisms used by MDSCs to inhibit T cell activity relies on cysteine homeostasis. Indeed, MDSCs and macrophages, but not T cells, express the cystine/glutamate transporter xCT (SLC7A11 or system X_c_^-^) on their surface, allowing them to import extracellular cystine, a major oxidized form of cysteine, but without releasing cysteine back to the TME (Figure 1). Through this action, they deprive T cells of cysteine that is necessary for their activity and function [59]; cystine import regulates the cellular cysteine levels of the T cell and maintains the reduced glutathione (GSH) pool that is necessary to counter oxidative stress.

In macrophages, ROS generated by succinate oxidation drives pro-inflammatory macrophage rewiring into an M1 phenotype and promotes the pro-inflammatory cytokine production [60]. However, in cancer, ROS-induced inflammatory signaling in TAMs and MDSCs favors tumorigenesis and metastasis. Liang et al. have used the diethylnitrosamine (DEN) mouse model of inflammation-driven liver cancer to demonstrate a key role of ROS and nicotinamide adenine dinucleotide phosphate (NADPH) oxidase (NOX)1 expression in macrophages in promoting liver tumorigenesis (Figure 2). Consistently, they have shown that the myeloid-specific deletion of *Nox1* resulted in fewer and smaller liver tumors [61]. ROS stimulation of inflammatory factors such as NF-κB can additionally induce immunosuppression through the up-regulation of PD-L1, immunosuppressive chemokines and angiogenic factors, as has been shown in response to the chemotherapeutic agent paclitaxel [62]. In response to the ROS production, the redox-sensitive transcription factor nuclear factor erythroid 2-related factor 2 (Nrf2) dissociates from Kelch-like ECH-associated protein 1 (KEAP1) and translocates to the nucleus to upregulate anti-oxidative [63,64] and anti-inflammatory [65] programs, including the induction of xCT1 [66,67] (Figure 2). The myeloid-specific deletion of *Nrf2* resulted in ROS accumulating in the MDSCs, and enhanced lung metastasis in the LLC mouse model [68], further confirming the deleterious impact of aberrant ROS signaling in myeloid cells on cancer progression.

## 4. Lipids and Cholesterol Metabolism Shape Immunosuppressive Myeloid Cells in the TME

Several studies have now confirmed a dependency of M2 macrophages, TAMs and MDSCs on lipid and cholesterol metabolisms. Inhibiting the lipid transport, lipolysis and FAO were shown to blunt their immunosuppressive and pro-tumoral activities. Mechanistically, the transcriptional activities of STAT6 downstream of IL-4, and of the peroxisome proliferator-activated receptor Gamma (PPAR*γ*) in conjunction with its co-activator PPAR*γ*-coactivator-1beta (PGC-1*β*), are key for the differentiation and activation of tumor-associated myeloid cells (Figure 2). Collectively, they function by upregulating genes involved in lipid uptake, e.g., CD36, mitochondrial biogenesis, OXPHOS and anti-inflammatory effectors. Initially, Chawla and coworkers demonstrated in vitro that PGC-1*β* was induced by STAT6 downstream of IL-4 signaling and that the uncoupling of mitochondrial respiration inhibited the expression of M2 genes such as ARG1. They also showed that the deletion of STAT6 or the downregulation of PGC-1*β* impaired the M2 macrophage program [69]. In the same system, Pearce and colleagues showed that CD36-mediated uptake of triacylglycerol substrates and their lipolysis in the lysosome via by lysosomal acid lipase (LAL) were important in driving FAO, and that the inhibition of lipases using the weight-loss agent Orlistat blunted the M2 macrophage markers expression and M2 protective responses in the *Heligmosomoides polygyrus* helminth infection mouse model [70]. In cancer, polyunsaturated fatty acids (PUFAs) promote the expansion and immunosuppressive function of MDSCs through ROS production and JAK-STAT3 activation [71] (Figure 2). In addition, it was observed that TAMs upregulate the storage of unsaturated fatty acids in lipid droplets [72,73] (Figure 1). Xiang et al. have shown that one mechanism leading to the lipid accumulation in TAMs is the downregulation of monoacylglycerol lipase (MGLL). The decreased MGLL expression stimulates cannabinoid receptor 2 (CB2) activity, which drives the pro-tumoral function of TAMs [74]. Similarly, MDSCs increase their fatty acid uptake, and mitochondrial mass and FAO inhibit this pathway with etomoxir, an irreversible inhibitor of carnitine palmitoyltransferase-1 (CPT-1), dampening their immunosuppressive function and subsequently leading to reduced tumor growth [75]. A recent study of note demonstrated that etomoxir inhibited M2 polarization independently of long chain FAO, which was found to be dispensable, but through the disruption of coA homeostasis [76]. The analysis of the FA transporter involved in the accumulation of triglycerides in PMN-MDSCs uncovered the fatty acid transport protein 2 (FATP2) as a regulator of PMN-MDSC immunosuppressive function [77]. The full body deletion of the FATP2-encoding gene *slc27a2*, or the PMN-specific deletion of this gene, led to an enhanced level of tumor rejection in mice. FATP2 acted by enhancing the uptake of arachidonic acid and mediated immunosuppression through prostaglandin E2 (PGE2) (Figure 2). The inhibition of FATP2 was shown to enhance the efficacy of immune checkpoint blockade in tumor-bearing mice [77]. One the mechanisms by which PGE2 exerts immunosuppression is through the upregulation of PD-L1 in TAMs and MDSCs; indeed, these cells were shown to express elevated levels of the enzymes upstream of PGE2 production, namely microsomal PGE2 synthase 1 (mPGES1) and Cyclooxygenase (COX)2, and their pharmacological inhibition dampened the PD-L1 expression [78]. An additional mechanism by which tumor cells promote the TAM function is by enhancing TAM membrane cholesterol efflux through ABC transporters, which reduces lipid rafts, inhibits IFN*γ*-induced gene expression and favors IL-4-induced macrophage polarization [79]. The myeloid-specific deletion of both *Abca1* and *Abcg1* transporters resulted in impaired TAM pro-tumoral functions and a reduced tumor growth in the ID8 mouse model of ovarian cancer [79]. Together, these studies propose several targets in the lipid metabolism pathway to thwart the immunosuppressive functions of myeloid cells in the TME.

## 5. Myelosuppressive Effects of Adenosine

A characteristic of the TME is the high levels of the immunosuppressive metabolite adenosine. Adenosine accumulates due to the rapid catabolism of ATP released by myeloid cells and dying tumor cells by the ectonucleotidases CD39 and CD73. CD39 converts ATP and ADP to AMP whereas CD73 converts the latter to adenosine. Adenosine can act directly on T cells to blunt their anti-tumoral activity through binding to its receptor, the A2A adenosine receptor (A2AR) [80,81]. The expression of A2AR on myeloid cells, including macrophages, DCs and MDSCs, also contributes to immunosuppression in the TME (Figure 1). This has been demonstrated with myeloid-specific deletion of *A2ar*, which resulted in enhanced T cells and NK cells of anti-tumoral activity and reduced melanoma metastasis in mice [82]. The adenosine generated by cancer cells is capable of stimulating macrophage migration, as demonstrated in an in vitro coculture system using ovarian cancer cell lines. In vivo, macrophages upregulate CD39 and CD73 expression in the TME, and CD73 expression is also provided by stromal fibroblasts [83]. Consistently, Wang et al. have reported that the adenosine released by tumor cells promotes macrophage proliferation in human HCC through the A2A pathway. The activated macrophages release GM-CSF which potentiates A2AR expression on macrophages, suggesting a synergistic effect of adenosine and GM-CSF to promote the macrophage proliferation in HCC [84]. Collectively, a blockade of adenosine production or signaling in the TME could be promising therapeutic strategies to improve anti-tumor immune responses.

## 6. Wnt Ligands Potentiate Myeloid Cells—Cancer Cells Metabolic Crosstalk

The Wnt pathway is an essential homeostatic pathway regulating several processes in embryogenesis and in adult tissues, notably cellular proliferation, migration and stemness, and is one of the central pathways upregulated in cancer. Besides driving tumorigenesis, the sustained activation of the Wnt pathway is also associated with a resistance to cancer therapies, as reviewed in [85]. Wnt signaling is engaged when one of 19 secreted Wnt ligands bind to members of the Frizzled receptors family, leading to the stabilization of *β*-catenin and downstream transcriptional responses. Macrophages have emerged as an important source of Wnt ligands, contributing to tumor growth and progression by driving the Wnt/*β*-catenin activation in cancer cells, as reviewed in [86]. In a feed-forward mechanism, tumor-cell-derived Wnt ligands promote the M2 polarization of macrophages. For instance, Yang et al. have shown that the depletion of Wntless from a hepatocellular carcinoma (HCC) cell line (Hepa1-6) inhibited M2-like TAMs in the TME following orthotopic transplantation, due to the impaired Wnt/*β*-catenin activation in TAMs [87]. Concordantly, Tian et al. have recently shown that through the upregulation of lncRNA (LINC00662), HCC cells secreted Wnt3A, which enhanced tumor growth in an autocrine manner and promoted a pro-tumoral TAM profile through the paracrine Wnt/*β*-catenin signaling in macrophages [88]. Other Wnt ligands have been implicated in tumor cells-myeloid cells crosstalk. For instance, MDSCs were reported to secrete Wnt5a and to depend on its autocrine signaling for full suppressive activity. The myeloid-specific *Wnt5a* deletion led to a decreased level of tumor growth in a mouse melanoma model, associated with diminished levels of intra-tumoral MDSC and Treg infiltration [89]. In a follow-up study, DeVito et al. reported that the inhibition of Wnt5a signaling enhanced the efficacy of immune checkpoint blockades in a mouse melanoma model, by thwarting tolerogenic DCs, decreasing Kynurenine levels and reducing PMN-MDSC infiltration in the TME [90]. These studies highlight the complexity of interactions among cells in the TME and the importance of onco-metabolic signals in immune evasion mediated by myeloid cells.

## 7. Clinical Trials Targeting Myeloid Cells, including via Their Metabolic Pathways

Myeloid cells are important targets for the next generation of immunotherapies. Several strategies have thus far been envisaged to deplete them, prevent their recruitment and modulate their activities. Below, we have highlighted a select few and direct the reader to recently published reviews [91,92]. The inhibition of the CSF1/CSF1R axis alone with antagonistic mAbs (NCT03336216, NCT02471716, NCT01444404) or in combination with immune checkpoint inhibitors (ICI) (NCT02777710, NCT03927105, NCT03153410) or other biologics, e.g., BRAF/MEK inhibitors (NCT03101254), has proven promising in early clinical trials of advanced solid tumors. However, this strategy has failed in some cancers such as glioblastoma [93] and triple negative breast cancer [94], presumably due to an inherent or acquired resistance. For instance, the effect of targeting monocyte-derived TAMs could be counteracted by higher suppressive neutrophils as demonstrated in a preclinical study [95]. In addition, grade 3 adverse effects have been reported [96], which have been attributed to the depletion of TRMs. The field is therefore moving away from an indiscriminate targeting of myeloid cells and much effort is instead being devoted to targeting macrophage effectors, including metabolic factors, to rewire their functions. IDO inhibitors, for example, have been tested in several trials but have failed to demonstrate clinical efficacy in monotherapy or beyond that observed with ICI [97]. Nonetheless, alternative approaches targeting IDO are currently being tested in multi-arm “basket” trials. These include peptide vaccines against IDO and PD-L1, alone or in combination with anti-PD-1 (NCT03047928, NCT05077709).

Targeting the immunosuppressive effects of adenosine on macrophages is also under intense investigation in several trials, as reviewed in [98]. Adenosine receptor antagonism is being tested as a monotherapy and in combination with other immunotherapies in various cancers such as NSCLC (NCT04791839), breast cancer, melanoma, colorectal cancer, ovarian cancer and renal cell carcinoma (NCT03629756). It is also being tested in combination with chemotherapy in gastroesophageal cancer and colorectal cancer (NCT03720678), or chemotherapy in combination with immunotherapies (NCT04660812, NCT03719326, NCT03846310). However, caution is warranted as the antagonists currently being tested inhibit both A2AR and A2BR adenosine receptors and are not specific to macrophages, which may result in off-target secondary effects.

## 8. Conclusions and Perspectives

Targeting the metabolism of immunosuppressive myeloid cells in the TME is a promising therapeutic strategy. As discussed in this review, cancer cells employ a range of means to co-opt the metabolism of tumor-associated myeloid cells to favor malignant progression. Besides depriving myeloid cells of the essential nutrients required for their effector functions, cancer cells release a panoply of metabolites, including lactate and adenosine, to attract myeloid cells to the TME and rewire their metabolism from an anti-tumoral to a pro-tumoral one. In addition to providing trophic functions, promoting angiogenesis and inhibiting cytotoxic T cells and NK cells tumorilytic activities, TAMs and MDSCs further support the metabolism of cancer cells by releasing metabolites, cytokines or vesicles containing lncRNAs that reinforce the Warburg effect. The identification of central metabolic nodes and effectors, such as metabolite transporters, metabolic enzymes, transcription factors, etc., provide an entry point to curb such metabolic interdependence. The recent works reviewed here provide compelling evidence for the potential in targeting the cellular metabolism to boost the efficacy of immunotherapies, which hopefully will translate to the clinic soon.

## Data Availability

Not applicable.
